# Serum Irisin Levels Are Inversely Correlated With the Severity of Coronary Artery Disease Confirmed by Coronary Angiography: A Comparative Cross-Sectional Study

**DOI:** 10.7759/cureus.41475

**Published:** 2023-07-06

**Authors:** Yousaf Tanveer, Unaizah Saif, Yizhe Lim

**Affiliations:** 1 Internal Medicine, Craigavon Area Hospital, Northern Ireland, GBR; 2 Internal Medicine, King Edward Memorial University, Lahore, PAK

**Keywords:** angiogram, coronary artery disease, predictive, severity, biomarker, serum, heart, cardiology, myokine, irisin

## Abstract

Introduction

Irisin, a newly discovered myokine, has been reported for its role in coronary artery disease (CAD), which is a major cause of mortality worldwide. Atherosclerosis is the primary cause of CAD. Irisin has been reported to reduce atherosclerosis by improving endothelial function and inhibiting inflammation via iNOS/NF-κB pathways. We sought to investigate the relationship between serum irisin levels and the severity of CAD that is confirmed with coronary angiography.

Methods

A comparative cross-sectional study was designed between the Chemical Pathology and Cardiology departments at KEMU/Mayo Hospital in Lahore, Pakistan. Patients were divided into group A with mild CAD (<50% stenosis) and group B with moderate-severe CAD (>50% stenosis). Serum was collected from venous blood, and irisin levels were analyzed by ELISA. Inclusion criteria: patients with stable CAD. Exclusion criteria: History of coronary artery bypass grafting (CABG), acute coronary syndrome (ACS), active or chronic infection, hepatic or renal dysfunction.

Results

The mean + SD age (years) of patients in group B (57.0±9.5) was significantly higher than group A (50.0±13.7). Irisin levels (μg/ml) were significantly higher in group A (15.3±4.6) than in group B (9.3±2.4). Irisin levels were significantly negatively correlated with the severity of CAD (% stenosis).

Conclusion

Serum irisin levels are low in patients with moderate to severe CAD, and they are negatively correlated with the severity of CAD (% stenosis).

## Introduction

Coronary artery disease (CAD) is a condition caused by atherosclerosis of the epicardial coronary arteries [1]. It is a major cause of morbidity and mortality worldwide [2]. CAD is responsible for about half of all cardiovascular deaths [1]. Its prevalence is 29% in Pakistan [1,3]. In 2019, 30% to 40% of all deaths in Pakistan were due to cardiovascular diseases (CVD). According to an estimation, mortality from cardiovascular disease will reach 23.4 million in 2030 [1].

Atherosclerosis is the primary cause of CAD in 90% of cases. Other causes are hypertension, vasculitis, hypoxia, and severe anaemia. Diabetes, dyslipidemia, hypertension, family history, obesity, sedentary lifestyle, and smoking are important risk factors responsible for CAD [2]. Lipid accumulation, endothelial dysfunction, inflammation, and reactive oxygen species (ROS) are important underlying mechanisms in the pathogenesis of atherosclerosis [4]. The role of many new biomarkers has recently been investigated for the underlying pathophysiology of CAD, like troponin T, I, and C, hs-cTn, and H-FABP [4].

Myokines are a type of cytokine that has autocrine, paracrine, and endocrine effects and is mostly secreted by skeletal muscle [5,6]. They are often used as biomarkers for CAD. Among them, myostatin and leukaemia inhibitory factors (LIF) are the most well-known. Recently, Irisin, a novel myokine, has been reported. It is generated by exercise, and it is a new and exciting area for researchers due to its role in CAD [6].

Endothelial dysfunction, which is characterized by reduced nitric oxide (NO) bioavailability, is the earliest pathologic event in many cardiovascular diseases and contributes significantly to the initiation and progression of vascular injury [7]. Irisin is known to improve endothelial function by enhancing NO phosphorylation in the adenosine 5’-monophosphate activated protein kinase (AMPK) eNOS pathway [8]. It has also been reported to impose a protective effect on blood vessels by reducing atherosclerosis and inhibiting cell apoptosis. Inhibiting inflammation and the ROS/p38 MAPK/ NF-κB signalling pathway [9].

We set out to study the relationship between serum irisin levels in patients with CAD. This is so we can better understand its relationship and potential future implications in clinical application.

## Materials and methods

A comparative cross-sectional study was designed between the Chemical Pathology Department of KEMU and the Cardiology Department of Mayo Hospital, Lahore Pakistan. The inclusion criteria are for patients with stable coronary artery disease and are not limited by age and gender. The exclusion criteria are patients that have had coronary artery bypass grafting (CABG), acute coronary syndrome (ACS), active infection, chronic inflammatory disease, severe hepatic dysfunction, as well as renal dysfunction.

Patients are selected by non-probability convenient sampling and are divided into 2 groups; mild CAD defined as stenosis of <50%, and moderate-severe stenosis, defined as stenosis >50%. The patients have already had their CAD confirmed using coronary angiography prior to any of our tests [10].

The sample size required for our study for a two-sided test was calculated with the equation outlined below:

n = (Z(1−α/2) + Z(1−β))^2^ σ^2^/(μ0 − μ 1)^2^

where, σ^2^ is the variance, Z(−α/2) is the confidence level 95% = 1.96, Z(1−β) is the power of test as 90%, µ_0_ is the population mean I = 161.24, µ_1_ is the population mean II = 217.25.

After informed consent, the demographic and relevant information of each patient was recorded in a study proforma. Five millilitres of venous blood was collected under aseptic conditions. Serum was separated from samples after clotting through centrifugation at 3000 rpm, and ELISA was performed on samples for irisin levels in two to three batches.

Data were entered into Statistical Package for Social Sciences version 26 (SPSS-26; SPSS, Inc., Chicago, IL). Quantitative variables like age and irisin levels will be presented as mean ± SD. Qualitative variables like gender, smoking, hypertension, and diabetes were presented as frequencies and percentages. For the comparison of irisin in two groups of patients (group A with mild coronary artery disease having coronary artery stenosis <50% on angiography and group B with moderate to severe coronary artery disease having coronary artery stenosis >50% on angiography), an independent sample t-test was applied. A p-value of ≤0.05 was taken as significant. Pearson correlation was applied to determine the relationship between serum Irisin levels and the severity of coronary artery disease (% stenosis).

## Results

A sample size of 62 patients (31 patients in each group, group A with mild coronary artery disease and group B with moderate to severe coronary artery disease) was estimated by using a 5% level of significance, 90% power of the test, and expected mean values of 161.24 ± 52.43 for group B and 217.25 ± 82.55 for group A [9].

**Figure 1:**
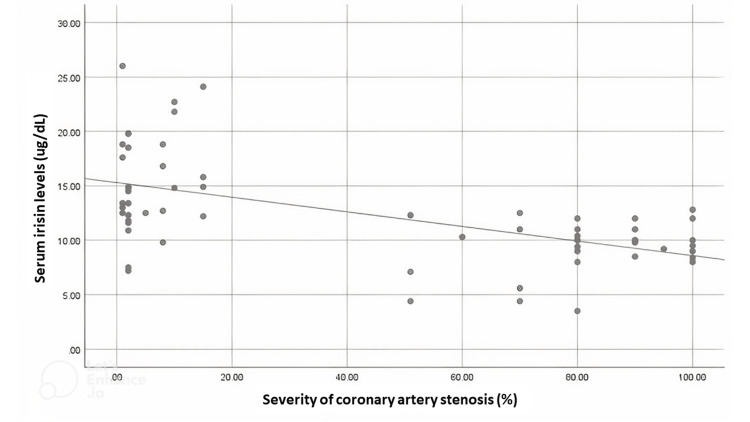
Scatter plot between severity of coronary artery stenosis (%) and serum irisin level (μg/ml).

The demographic and clinical characteristics are summarised in Table 1. The irisin levels concerning sex and severity of stenosis on angiography are summarised in Table 2. A scatter plot was created, and it shows a moderately negative relationship between the severity of coronary artery stenosis and serum irisin levels, illustrated in Figure 1.

**Table 1:** Demographic and clinical characteristics of patients in study groups.

Variables	Group A (n=31)	Group B (n=31)	p-value
Age (mean±SD)	50.0 ± 13.7	57.0 ± 9.5	*0.02
Sex (M/F)	9:22 (29%:71%)	28:3 (90%:10%)	*<0.05
Body mass index (mean±SD)	26.8 ± 7.0	26.8 ± 4.0	0.95
History of hypertension	18 (58%)	20 (65%)	0.6
History of smoking	7 (23%)	16 (52%)	*0.01

**Table 2:** Severity of stenosis of coronary arteries, and serum irisin levels with respect to sex.

Variables	Group A	Group B	p-value
Severity of coronary artery stenosis on angiography (%)	5.12 ± 4.91	82.0 ± 15.0	*<0.05
Serum Irisin levels (μg/ml)	15.3 ± 4.6	9.3 ± 2.4	*<0.001
Serum irisin levels in males (μg/ml)	16.2 ± 4.7	9.4 ± 2.5	0.54 between group A, and 0.79 between group B
Serum irisin levels in females (μg/ml)	14.9 ± 4.6	9.7 ± 1.1	
Correlation (r) between irisin levels and severity of coronary artery stenosis	−0.576		*<0.001

## Discussion

The ages of the patients in group A were significantly different from those in group B. This is in accordance with a study by Efe et al., which showed that there was a significant difference in the age of the patients in the two groups [6]. But the findings of our study are contrary to those of Deng and Klatt, which did not show any significant differences [2,11].

There were significantly more males than females in group B than in group A, showing that moderate to severe CAD was more common in males than females in our study subjects. Males have a generally greater tendency to develop atherosclerosis than females because premenopausal women have protection against atherosclerosis due to oestrogen [12]. The findings of our study are not in accordance with those of Efe et al. and Deng, in which both groups were matched for gender [2,11]. This difference might be attributed to patient ethnicity, as these studies were carried out on Chinese and Turkish populations, which were different from our study.

There were no statistically significant differences in body mass indices (BMI) between patients in groups A and B. This is in accordance with the study conducted by Deng [2], Efe et al. [6], and Klatt [11], where BMI was not statistically significant because both groups were matched for BMI.

The frequency of hypertension between the two groups was not statistically different (p=0.60), similar to Efe et al., in which the history of hypertension was not statistically significant in both groups [6]. Deng also reported similar results, with no difference in both systolic and diastolic blood pressure [2].

The frequency of smoking between the two groups was significantly different (p=0.01). There were mostly females in group A with mild CAD compared to group B, which had more males. This difference can be due to Pakistani culture, where women generally do not smoke. Those who smoke have a predisposition towards developing atherosclerosis [12], supported by Deng [2] but not in the study by Efe et al. [11].

The irisin levels were significantly lower in patients with moderate to severe stenosis in the coronary arteries than in patients with mild stenosis. Our results are comparable to a study by Efe et al. [6], which showed that patients with more severe stable CAD had significantly lower serum irisin levels than patients with less severe CAD [7]. The difference between our studies is that they used validated severity scores like the SYNTAX score.

This is also supported by Deng [2], who used a graded severity of CAD based on angiography called the Coronary Atherosclerosis Index (CAI) [13]. Klatt has a similar study design and results to ours, where those with CAD on percutaneous coronary intervention compared to a control group have lower serum irisin levels [11]. Our studies used coronary angiography to classify severity, which is often touted as the gold standard in the evaluation of CAD.

In terms of coronary artery stenosis, we have shown a statistically significant negative correlation between the groups. This is in accordance with published evidence [2,6,11]. Therefore, it can be concluded that irisin levels are lower in patients with more significant CAD based on both validated severity scores as well as angiographic findings, and irisin levels can therefore be used to predict the severity of stable CAD.

The role of irisin in the development of CAD is still unclear. The first proposed hypothesis for the role of serum irisin levels in CAD is that lower irisin levels cause endothelial dysfunction and reduced coronary blood flow due to reduced nitric oxide (NO) bioavailability. Irisin is thought to enhance NO phosphorylation via the AMPK-eNOS pathway and improve endothelial function [11]. Han et al. reported that systemic administration of irisin protected against endothelial injury and ameliorated atherosclerosis by inhibiting oxidative stress. Furthermore, decreased plaque area and lower levels of inflammation characterised by reduced infiltrating macrophages and T lymphocytes in the plaques were also observed [14]. Strasser, through systemic irisin administration in an animal study, reported improved endothelial function [15] and induced relaxation in mesenteric arteries [9]. Similarly, Deng showed that irisin treatment in obese mice and human umbilical vein endothelial cells both led to increased NO secretion and phosphorylation of AMPK [2]. Other animal studies, particularly in obese animals, demonstrated improved endothelial function when irisin was administered exogenously by enhancing NO phosphorylation through the AMPK-eNOS pathway [15,16]. Furthermore, through the inhibition of a separate NF-kB/iNOS pathway, irisin alleviates endothelial dysfunction in type-2 diabetes mellitus [17]. This is achieved through the reduction of oxidative and nitrative stresses and may serve as a pharmacological target in the prevention of microvascular complications of diabetes mellitus [17].

The other hypothesis suggests that lower irisin levels are a result of reduced coronary blood flow rather than a cause. According to this hypothesis, oxidative stress regulates the expression of irisin [18,19], and irisin is an important determinant of energy homeostasis, which positively correlates with skeletal muscle mass [20,21]. Specifically in the heart, irisin increases myocardial cell metabolism, inhibits cell proliferation, and promotes cell differentiation in vitro [22]. In animal models with ischemic cardiomyopathy after a myocardial infarct, in vitro incubation of myotubes suggests that decreased irisin levels are due to inflammatory cytokines [23]. This has been proposed to act as a protective mechanism, either by preserving energy homeostasis or by regulating muscle atrophy.

This study was cross-sectional and conducted on a small scale. Future studies are needed to assess improvements in patients’ clinical conditions by increasing irisin levels. Moreover, studies are needed to unveil the role of low irisin levels in CAD, whether they are a cause or a result of CAD. Elucidating the correct mechanism would lead to a better understanding of the clinical role of irisin [18]. Increasing irisin levels by exercise, pharmaceutical intervention, or recombinant irisin might help improve CAD by improving endothelial function.

## Conclusions

In our study, we have shown that in patients with stable coronary artery disease, increasing disease confirmed on coronary angiography is inversely correlated with serum irisin levels. This result, in combination with other studies with severity indices, provides conclusive results.

Although irisin’s role is unclear in coronary artery disease, early studies suggest irisin has a protective role in endothelial function. This is supported by improved endothelial function by systemic administration, suggesting targets for the development of future pharmaceuticals.
